# Establishment of a *STING*-Deficient HepG2 Cell Line through CRISPR/Cas9 System and Evaluation of Its Effects on *Salmonella* Replication

**DOI:** 10.1155/2024/9615181

**Published:** 2024-09-12

**Authors:** Lanqing Sun, Kai Huang, Xuan Huang

**Affiliations:** ^1^ Department of Laboratory Medicine Affiliated Hospital of Jiangnan University, Wuxi, Jiangsu, China; ^2^ Orthopaedic Institute Wuxi Ninth People's Hospital Affiliated to Soochow University, Wuxi, Jiangsu, China

## Abstract

**Background:**

*Salmonella enterica* serovar Typhimurium (*Salmonella* Typhimurium) is a common food-borne pathogen that causes gastroenteritis and can lead to life-threatening systemic disease when it spreads to vital organs, such as the liver. Stimulator of interferon genes (STING) is a crucial regulator of the host's innate immune response to viral infections, while its role in bacterial infections remains controversial. This study aims to establish a *STING*-deficient HepG2 cell line through the CRISPR/Cas9 system and evaluate its effects on *Salmonella* replication.

**Methods:**

In this study, a *STING* knockout HepG2 cell line was constructed through the application of CRISPR/Cas9 technology. We assessed cell viability and proliferation using the CCK-8 assay. Subsequently, we investigated the effect of *STING* deletion on *Salmonella* replication and the expression of type I interferon-related genes.

**Results:**

The *STING* knockout HepG2 cell line was successfully constructed using the CRISPR/Cas9 system. The proliferation capability was diminished in *STING*-deficient HepG2 cells, while *Salmonella* Typhimurium replication in these cells was augmented compared to the wild-type (WT) group. Following *Salmonella* infection, the transcriptional responses of type I interferon-related genes, such as *IFNB1* and *ISG15*, were inhibited in *STING*-deficient HepG2 cells.

**Conclusions:**

We successfully constructed a *STING*-deficient cell line. Our finding of increased *Salmonella* Typhimurium replication in *STING*-deficient HepG2 cells provides the basis for further studies on pathogen-host interactions.

## 1. Introduction

*Salmonella* infections are prevalent globally and continue to pose significant public health challenges in many developing nations. *Salmonella enterica* serovar Typhimurium (*Salmonella* Typhimurium) is a Gram-negative, facultative intracellular bacterial pathogen that can lead to severe gastroenteritis and systemic inflammation in humans and animals [1]. Additionally, it serves as a widely used model organism to investigate the molecular mechanisms involved in bacterial pathogenesis and pathogen-host interactions. As one of the most widespread pathogens transmitted through food and water, *Salmonella* Typhimurium often infiltrates the gastrointestinal barrier, resulting in symptoms like diarrhea and acute inflammation. Later in the infection process, it can disseminate to the mesenteric lymph nodes and may even spread to extraintestinal tissues, such as the liver [2]. Severe hepatic involvement with an acute hepatitis-like clinical appearance has been reported as a possible consequence of *Salmonella* infection [3, 4]. However, the molecular mechanisms of how hepatocytes respond to *Salmonella* infection remain incompletely defined.

Type I interferons (IFNs) are involved in the host response to *Salmonella* infection. Autophagy is a significant antimicrobial response that inhibits *Salmonella* growth [5]. IFN-*β* can be induced by autophagy through Toll-like receptors (TLRs) and the TRIF pathway, providing a protective effect on the host [6]. However, another study found that IFN-*β* increased host susceptibility to infection by restricting innate immune responses [7]. Besides autophagy, stimulator of interferon genes (STING) is one of the main activators of IFN-*β* production. The rapid detection of microbial agents is crucial to effectively trigger the host's defense mechanisms against infection. STING is an endoplasmic reticulum (ER)-associated membrane protein known to trigger type I IFN and proinflammatory responses upon the detection of cytosolic DNA by cyclic GMP-AMP synthase (cGAS) [8]. The cGAS-STING pathway is an important cytosolic surveillance pathway (CSP), and increasing evidence demonstrates its essential role in host defense against a variety of bacterial pathogens [9–12]. Moreover, STING is also a direct sensor of cyclic dinucleotides (CDNs) generated by numerous intracellular bacteria [13]. Although the cGAS-STING pathway has been extensively studied for its role in promoting the antiviral innate immune response, its function in bacterial infections remains debatable. Therefore, constructing *STING* knockout cell lines is helpful for clarifying the role of STING in *Salmonella* infection.

HEK293T and Vero-E6 cells are known to have deficiencies in cGAS-STING signaling [14]. We aimed to investigate the cellular response to *Salmonella* infection in both STING-containing and STING-deficient systems, focusing on hepatocytes. Therefore, we set out to delete STING in cells that have demonstrated STING activation through gene knockout. As previously reported, STING is not expressed in primary human hepatocytes and Huh7 cells [15]. However, researchers have detected STING expression and cGAS-STING signaling activation in another human hepatocyte-derived cell line, HepG2 [15, 16].

The clustered regularly interspaced short palindromic repeats/CRISPR-associated protein 9 (CRISPR/Cas9) system is a recently developed and highly efficient genome editing technology [17]. Guided by gRNA, Cas nucleases orchestrate the precise cleavage of DNA double strands, playing a crucial role in gene editing. This method offers benefits such as convenience, efficiency, cost-effectiveness, and ease of use [18]. In this study, we constructed a *STING* knockout HepG2 cell line using CRISPR/Cas9 technology. Subsequently, we investigated the effect of *STING* deletion on *Salmonella* replication. The generated knockout cell line represents an optimal resource for subsequent investigations into *Salmonella* infection.

## 2. Materials and Methods

### 2.1. Cell Culture

The wild-type (WT) HepG2 cell line was kindly provided by Professor Qiaoming Long (Medical College of Soochow University, Suzhou, China) [19]. Cells were incubated at 37°C with 5% CO_2_ in Dulbecco's Modified Eagle's medium (DMEM) (SH30243.01B; Hyclone) supplemented with 10% fetal bovine serum (FBS; 04-001-1ACS; Biological Industries) and 1% Penicillin-Streptomycin Solution (C0222; Beyotime Biotechnology).

### 2.2. Bacterial Strain and Infection

*Salmonella* Typhimurium strain SL1344 in this study was kindly provided by Professor Qian Yang (Nanjing Agricultural University, Nanjing, China). *Salmonella* Typhimurium strain was grown in Luria Bertani (LB) medium at 37°C with shaking overnight. The following day, it was diluted at a ratio of 1 : 100 with fresh LB medium and then cultured until it reached the logarithmic phase of growth. Upon washing 3 times with sterile PBS, bacterial numbers were estimated and adjusted based on the optical density at 600 nm absorbance and ready for the further experiments. For infection, bacteria were washed in PBS and subsequently resuspended in DMEM at a multiplicity of infection (MOI) of 10.

### 2.3. Design sgRNA

The *STING* gene sequence was determined by GRCh38 human reference genome. sgRNAs were designed using an online tool named Wellcome Sanger Institute Genome Editing (WGE) (https://wge.stemcell.sanger.ac.uk/search_by_seq). The CRISPR/Cas9 system specifically targeted exon 6 of the *STING* genome. After screening by specificity and efficiency score, sgRNAs were chosen as Table 1.

### 2.4. Construction of sgRNA Plasmid

DNA fragment containing the U6 promoter and sgRNAs was amplified by polymerase chain reaction (PCR) using PUC57-sgRNA-U6 plasmid as a template with the primers in Table 2. Then, PCR products and primary pGL3-U6-2sgRNAs plasmids were digested with Esp3I enzyme (ER0451; Thermo Scientific) and ligated with T4 DNA ligase (2011A; Takara) according to manufacturer's instructions.

### 2.5. Construction of *STING* Knockout Monoclonal Cell Line

Cells were transfected with constructed pGL3-U6-2 sgRNAs together with pSt1374-N-NLS-Flag-Cas9 plasmids using the Lipofectamine™ 3000 Transfection System (L3000001; Thermo Scientific) according to manufacturer's instructions. After 48 hours of transfection, cell screening was conducted using a puromycin concentration of 2 *μ*g/mL. The residual cell population was subjected to single-cell separation using a limited dilution method and transferred into a 96-well plate. Monoclonal identification was performed after 2 weeks of cell culture.

### 2.6. PCR and Sanger Sequencing of *STING* Knockout Cell Line

Monoclonal cells were validated through PCR followed by Sanger sequencing. Total cellular DNA was extracted using FastPure Cell/Tissue DNA Isolation Mini Kit (DC102; Vazyme). PCR reaction was performed according to the protocol of 2 × Phanta Max Master Mix (P515; Vazyme) with the primers in Table 3. Then, the PCR reaction settings were set up as follows: initial denaturation 95°C for 3 min, 30 cycles of denaturation 95°C for 15 s, annealing 55°C for 15 s, 72°C extension for 1 min, and 72°C extension for 5 min. Next, the PCR product was purified using electrophoresis and FastPure Gel DNA Extraction Mini Kit (DC301; Vazyme). Sanger sequencing was performed at GENEWIZ Co., Ltd. (Suzhou, China).

### 2.7. Western Blotting Analysis

The total protein of WT or *STING* knockout HepG2 cells were extracted using RIPA Lysis Buffer (R0278; Sigma Aldrich) containing the Protease Inhibitor Cocktail (4693132001; Roche). Protein concentrations were measured with BCA Protein Assay Kit (P0010; Beyotime Biotechnology). Protein sample was separated by SDS-PAGE electrophoresis and then transferred to polyvinylidene difluoride (PVDF) membranes. Membranes were blocked and incubated with STING antibody (19851-1-AP; Proteintech) and *β*-actin (SAB1305554; Sigma-Aldrich) at 4°C overnight. Then, membranes were incubated with the HRP-conjugated antibody at room temperature for 1 h. Finally, the blots were visualized with an ECL luminescence reagent (ma0186; Meilunbio).

### 2.8. Cell Proliferation Analysis

CCK-8 Cell Counting Kit (A311; Vazyme) was used to perform cell proliferation assays. In this procedure, 2 × 10^4^ HepG2 WT or *STING* knockout cells were seeded in 96 well plates and added with 10 *μ*L of the CCK-8 reagent for another 1 h, 2 h, 3 h, and 4 h. Optical density was measured at 450 nm.

### 2.9. Bacterial Replication Analysis

For the assessment of bacterial replication, 4 × 10^5^ HepG2 cells were initially seeded into 12 well plates and then infected with SL1344 at an MOI of 10 the next day. The amount of bacterial suspension added to the cell cultures was calculated from growth curves measured in the laboratory before. After a 0.5 h infection, the cells were washed with PBS and subsequently cultured for an additional 0.5 h in DMEM-FBS (10%) supplemented with 100 *μ*g/mL gentamicin (A620217; Sangon Biotech) to remove extracellular bacteria. Then, cells were lysed with 0.3% Triton X-100 (V900502; Sigma-Aldrich), diluted cell lysates were plated onto *SS* agar culture plates, and the cultures were incubated at 37°C overnight to enumerate the internalized bacteria [20].

### 2.10. RNA Isolation and Quantitative Real-Time PCR (qPCR)

Cells were lysed, and total RNA was extracted using TRIzol reagent. RNA was reverse transcribed by RT Reagent Kit (RR047A; Takara). qPCR was performed using SYBR Green Supermix kit (1725121; BIO-RAD) on CFX96 Touch Real-Time PCR Detection System (BIO-RAD). Primers are shown in Table 4.

### 2.11. Statistical Analysis

The analysis of experimental data was carried out by GraphPad Prism software (La Jolla, CA, USA) and IBM SPSS Statistics for Windows (Armonk, NY, USA). Statistical comparisons between two datasets were executed utilizing the unpaired Student's *t*-test. One representative dataset of at least three independent experiments was shown as the mean ± standard error of the mean (SEM).

## 3. Results

### 3.1. Construction of STING Knockout HepG2 Cell Strain

**Figure 1:**
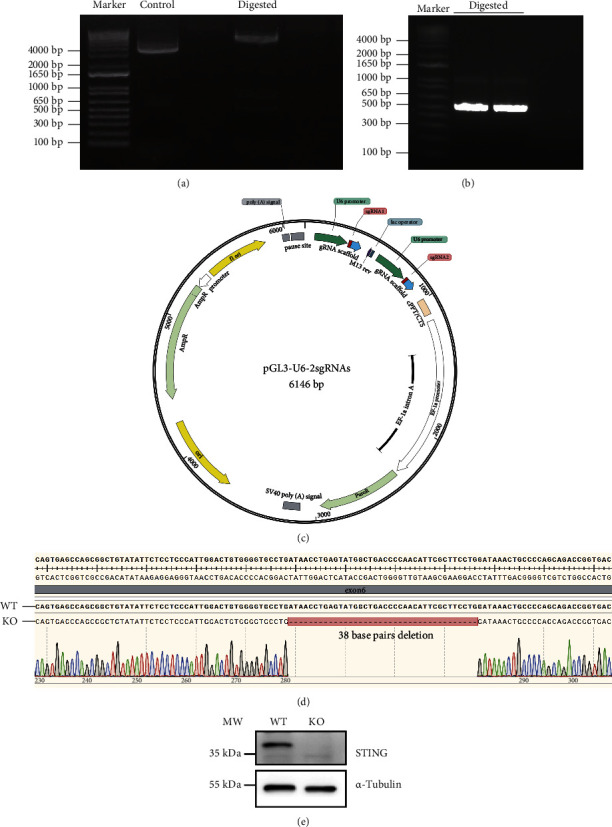
Construction of *STING* knockout HepG2 cell strain. (a) Agarose gel electrophoresis of isolated plasmids, including nondigested primary pGL3-U6-2sgRNAs plasmids (control) and plasmids digested by Esp3I restriction enzyme (digested). (b) Agarose gel electrophoresis of PCR products containing the U6 promoter and sgRNAs targeting exon 6 of human *STING* gene and digested by Esp3I restriction enzyme. (c) Constructed pGL3-U6-2sgRNAs plasmid, sgRNA, and scaffold sequence located downstream of the U6 promoter, and puromycin coding sequence located downstream of EF-1 alpha promoter. (d) DNA sequence analysis of WT and knockout cell line showing the *STING* mutation, the red box indicates the deleted 38 base pairs. (e) Western blotting analysis of *STING* protein in WT and *STING* knockout (KO) HepG2 cell lines.

pGL3-U6-2sgRNAs plasmids and the PCR products containing the U6 promoter and sgRNAs were digested by Esp3I enzyme (Figures 1(a), 1(b)). After ligation, the combinant plasmid containing two sgRNAs targeting exon 6 of the *STING* gene was constructed (Figure 1(c)). The recombinant and Cas9 expression plasmids were cotransfected into HepG2 cells, followed by puromycin screening and single cell separation. After monoclonal cell formation, total DNA of the cells was isolated, and PCR reaction was performed with primers designed on the exon 6 of human *STING* (Table 3). Sanger sequencing of the PCR products revealed that 38 nucleotides of exon 6 were missing, leading to a frameshift mutation in human *STING*, indicating that *STING* gene was knocked out successfully (Figure 1(d)). When passaged at a proportion of 1 : 3, both *STING* knockout and WT cell lines achieved 90% confluence within 3 days. Western blotting analysis showed that protein levels were undetectable in the *STING* knockout cell line compared to the WT cell line, further confirming the efficiency of the gene knockout (Figure 1(e)).

### 3.2. *STING* Knockout Suppresses HepG2 Cell Proliferation

**Figure 2:**
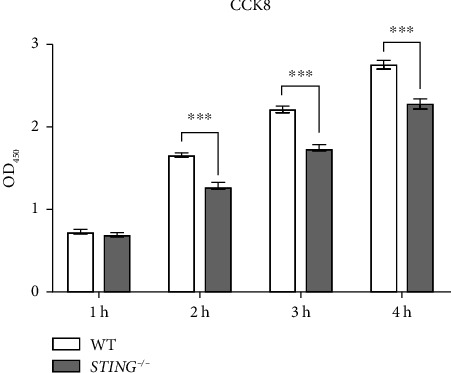
*STING* knockout suppresses HepG2 cell proliferation. The proliferation ability of WT and *STING* knockout HepG2 cells. Cells were seeded in 96 well plates, and the optical density was assessed at the indicated time intervals subsequent to the addition of the CCK-8 reagent at 450 nm. Data are expressed as the mean ± SEM, and statistically significant differences are indicated. ^∗∗∗^*P* < 0.001.

We then used the CCK-8 kit to examine how *STING* knockout affects the proliferation of the HepG2 cell line. Our results showed that the optical density (O.D.) values of the *STING* knockout group were significantly lower compared to the WT group at several time points after incubation with the CCK-8 reagent (Figure 2). This finding indicates that *STING* knockout inhibits the proliferation ability of HepG2 cells.

### 3.3. *STING* Knockout Promotes *Salmonella* Replication

**Figure 3:**
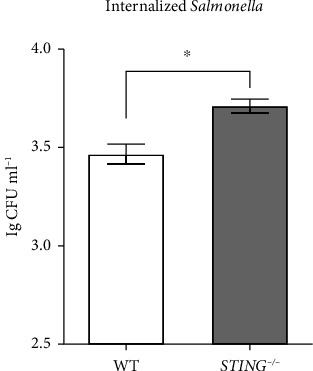
*STING* knockout promotes *Salmonella* replication. The *Salmonella* replication in WT and *STING* knockout HepG2 cells. Cells were seeded in 12 well plates and infected with *Salmonella* Typhimurium at an MOI of 10 for 1 h. The internalized bacteria were enumerated by plating. Data are expressed as the mean ± SEM, and the statistically significant difference is indicated. ^∗^*P* < 0.05.

To investigate the effect of *STING* knockout on *Salmonella* replication in the HepG2 cell line, internalized bacteria numbers was analyzed after infection at an MOI of 10 for 1 h. The results revealed that *Salmonella* replication in the HepG2 cells with *STING* knockout was significantly higher compared to the WT group (Figure 3). This finding indicates that STING plays a negative role in *Salmonella* replication.

### 3.4. *STING* Knockout Suppresses Type I IFN Response during *Salmonella* Infection

**Figure 4:**
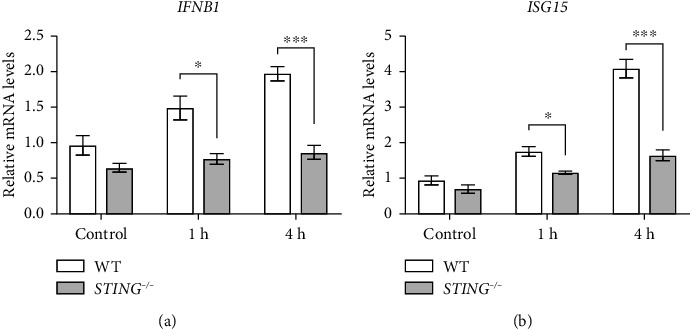
*STING* knockout suppresses type I IFN response during *Salmonella* infection. The qPCR analysis of *IFNB1* (a) and *ISG15* (b) gene expression. WT and *STING* knockout HepG2 cells were infected with SL1344 at an MOI of 10 for 1 or 4 h (*n* = 4). Data were normalized to the WT uninfected control group (set as 1). *ACTIN* was used as the housekeeping gene. Data are expressed as the mean ± SEM, and statistically significant difference is indicated. ^∗^*P* < 0.05; ^∗∗∗^*P* < 0.001.

*Salmonella* induces a type I IFN response in macrophages, which depends on the cGAS-STING pathway [21]. To investigate the type I IFN response in hepatocytes following *Salmonella* infection, we examined the expression of IFNB1 and interferon-stimulated gene 15 (ISG15). Our results showed that in WT HepG2 cells, the mRNA levels of both IFNB1 and ISG15 increased following *Salmonella* infection but were significantly lower in the *STING* knockout group compared to the WT group (Figures 4(a), 4(b)). These findings, combined with the increased *Salmonella* replication observed in the *STING* knockout group, suggest that the type I IFN response plays a role in defending against *Salmonella* infection.

## 4. Discussion

*Salmonella* Typhimurium is a bacterial pathogen that causes various diseases in humans and animals. In severe cases of *Salmonella* Typhimurium infection, the bacteria can enter the bloodstream (bacteremia) and spread to various organs, including the liver. This can lead to disseminated infection and systemic symptoms [1]. STING is primarily known for its role in regulating the immune response against viral infections and certain intracellular pathogens. Some studies suggest that STING may also play a role in controlling bacterial infections by modulating inflammation and immune responses [8]. However, the interaction between *Salmonella* Typhimurium—a food-borne bacterial pathogen—and STING has not been studied as extensively as its interaction with viruses, particularly in nonphagocytic cells such as hepatocytes. Among common hepatic cell lines, HepG2 has been used as an *in vitro* model to investigate the cellular and molecular mechanisms involved in *Salmonella* infection and its impact on hepatocytes [22, 23]. Additionally, while STING expression is not detected in mouse and human primary hepatocytes, it is expressed and functional in HepG2 cells [15, 16]. In this study, we generated a *STING*-deficient HepG2 cell line using the CRISPR/Cas9 system, an efficient and widely used tool for generating knockout cell lines. We found that *STING* knockout cells are more susceptible to *Salmonella* infection, with increased bacterial growth. This indicates that STING exerts an antibacterial effect in HepG2 cells.

Chronic inflammation resulting from persistent activation of the cGAS-STING pathway is strongly associated with cancer progression [24, 25]. In hepatocellular carcinoma tissues, the mRNA levels of genes involved in the cGAS-STING pathway are up-regulated [16]. HepG2 is an immortal cell line derived from a hepatocellular carcinoma patient, and chromosomal instability (CIN) is a hallmark of human cancer [26]. In our study, we found that *STING* knockout inhibited the proliferation ability of HepG2 cells. The reduced cellular viability observed in HepG2 cells following *STING* deletion aligns with recent research, which suggests that the cGAS-STING pathway drives the survival of chromosomally instable cancers [27]. One potential mechanism by which STING may suppress *Salmonella* Typhimurium replication is through the production of type I interferons and proinflammatory cytokines. The role of IFN-*β* in *Salmonella* infection remains ambiguous. In macrophages, IFN-*β* has been shown to both promote and inhibit innate immune responses, such as the release of inflammatory cytokines following *Salmonella* infection [6, 7]. Upon sensing bacterial DNA, STING can activate the production of interferons, which initiates an antiviral response [28, 29]. Although *Salmonella* Typhimurium is a bacterium, the antiviral response may still contribute to immune defense against pathogens. Our data showed that the mRNA level of IFNB1 was reduced in *STING* knockout cells, suggesting an antibacterial function of IFN-*β* in the HepG2 cell model of *Salmonella* infection. Further experiments are needed to manipulate IFN-*β* levels in this model to confirm its antibacterial role. ISG15 is an interferon-induced protein that aids in clearing invading bacteria [30]. We observed a significant decrease in ISG15 mRNA expression in the *STING* knockout group, combined with increased *Salmonella* replication, suggesting that ISG15 may have an anti-infective role in HepG2 cells. In addition to type I IFN signaling, bacterial infection can directly activate ISG15 expression through the STING pathway [31]. Therefore, STING deletion alone in *Salmonella*-infected HepG2 cells may significantly contribute to the downregulation of ISG15 expression. Another possible pathway involves the interaction between STING and pattern recognition receptors (PRRs), such as TLRs [32, 33]. Activation of TLR can enhance STING signaling pathway, potentially boosting host immune responses [34]. During *Salmonella* infection, autophagy plays a critical role in inhibiting bacterial survival [35]. STING has been reported to directly activate autophagy to regulate the innate immune response [36, 37]. Thus, autophagy may be involved in STING-mediated restriction of *Salmonella* survival in HepG2 cells. The detailed mechanisms underlying our findings warrant further investigation.

In conclusion, we constructed a *STING* knockout HepG2 cell line using the CRISPR/Cas9 system. Our findings reveal that *STING* deletion inhibited HepG2 cell proliferation and facilitated *Salmonella* Typhimurium replication. It is important to note that our understanding of STING's role in bacterial infections, including *Salmonella*, is still developing. The established cell line serves as an effective tool for further research on *Salmonella* infection.

## Figures and Tables

**Table 1:** sgRNA sequence.

sgRNA	Sequence 5′-3′
sgRNA-1	TATCCAGGAAGCGAATGTTGGGG
sgRNA-2	CGGTGACCATGCTGGCATCAAGG

**Table 2:** PCR primers.

Primer	Sequence 5′-3′
F	ATGCGTCTCAACCGTATCCAGGAAGCGAATGTTGGTTTTAGAGCTAGAAATAGCAAG
R	ATGCGTCTCGAAACTGATGCCAGCATGGTCACCGCGGTGTTTCGTCCTTTCCACAAG

**Table 3:** PCR primers.

Primer	Sequence 5′-3′
F	AAAGGGTGGTGCAGTAAGAGG
R	GGGCAGCTTTATGGTCAAGAG

**Table 4:** qPCR primers.

Primer	Sequence 5′-3′
*IFNB1*-F	CTTGGATTCCTACAAAGAAGCAGC
*IFNB1*-R	TCCTCCTTCTGGAACTGCTGCA
*ISG15*-F	CTCTGAGCATCCTGGTGAGGAA
*ISG15*-R	AAGGTCAGCCAGAACAGGTCGT
*ACTIN-*F	CACCATTGGCAATGAGCGGTTC
*ACTIN-*R	AGGTCTTTGCGGATGTCCACGT

## Data Availability

All data are available in the paper.

## Abbreviations

*Salmonella* Typhimurium: *Salmonella enterica* serovar Typhimurium
cGAS: Cyclic GMP-AMP synthase
CRISPR/Cas9: The clustered regularly interspaced short palindromic repeats/CRISPR-associated protein 9
CSP: Cytosolic surveillance pathway
DMEM: Dulbecco's Modified Eagle's medium
ER: Endoplasmic reticulum
FBS: Fetal bovine serum
IFN: Interferon
ISG15: Interferon-stimulated gene 15
KO: Knockout
LB: Luria Bertani
MOI: Multiplicity of infection
PCR: Polymerase chain reaction
PRR: Pattern recognition receptor
SEM: Standard error of the mean
STING: Stimulator of interferon genes
TLR: Toll-like receptor
WGE: Wellcome Sanger Institute Genome Editing
WT: Wild-type.
